
JOURNAL INFORMATION
==============================
NLM Title Abbreviation: HPB Surg
NLM Journal ID: 9002972

HPB Surgery

ISSN: 0894-8569
EISSN: 1607-8462
Publisher: Wiley

ARTICLE INFORMATION
==============================
PMCID: PMC2423496
DOI: 10.1155/1988/62630
Article ID: S0894856988626300
Article version: 1
Subjects: Research Article

HPB News

Print publication date: 1988
Volume: 1
Issue: 1
First page: 91
Last page: 92
Copyright: Copyright © 1988 Hindawi Publishing Corporation.
Copyright year: 1988
Copyright holder: Hindawi Publishing Corporation
License: This is an open access article distributed under the Creative Commons Attribution License, which permits unrestricted use, distribution, and reproduction in any medium, provided the original work is properly cited.
License URL: http://creativecommons.org/licenses/by/

==============================
*HPB Surgery* 1988, Vol. 1, pp. 91–92
Reprints available directly from the publisher
Photocopying permitted by license only

© 1988 Harwood Academic Publishers GmbH
Printed in Great Britain

### HPB NEWS

### IBA MEETING IN NICE, SEPTEMBER 12–14, 1988

IBA, previously International Biliary Association having had its name recently changed to International Hepato-Biliary-Pancreatic Association is holding its annual meeting in Nice, September 12–14, 1988. The organizer of the meeting is Professor Jean Mouiel, Hôpital St. Roch, 5 rue Pierre, Devolue, 60000 Nice, France. The program will contain lectures on intra-hepatic lithiasis (J. Wong, Hong Kong), lithotripsy in biliary diseases (G. Baumgartner, FRG), Alveolar echinococcos (M. Gilette, France) and endocrine tumors of the pancreas (H.T. Debass, USA). In addition, about 10 table discussions are arranged on topics such as hepato-cellular carcinoma, sclerosing cholangitis, chronic pancreatitis and liver transplantation. The meeting is open for free papers, posters and videofilms in the areas of hepatic, pancreatic and biliary surgery.

### NEWS FROM GREEK HPB CHAPTER

A HPB chapter was formed in Greece about 1 year ago. At the autumn meeting in Athens, Stig Bengmark from Lund and Henri Alexandre were the guest speakers.

A second meeting was arranged April 29–May 1 in Alexandreopolis. The main topic for the meeting was what is new in biliary surgery.

### NEWS FROM THE ARGENTINE CHAPTER

The Argentine chapter of the World Association of HPB Surgery had its first meeting, April 24–28 in La Plata, Buenos Aires. President of the meeting was Professor Osvaldo H. Mammoni. Among the guest speakers were Stig Bengmark, Lund, Claude Couinaid, Paris, G.M. Gazzaniga, Genova, Marcel Macado, San Paulo, Paul McMaster, Birmingham and Louise Pradere, Uruguay. Around 100 participants attended the meeting which continued for 4 days. In addition to the guest lectures, panel discussions and free papers were arranged and videos were shown.

The meeting was held in excellent facilities of a new hospital; Centro Oncologico De Excellencia M.B. Gonnett, which was founded by the internationally well-known HPB and oncology surgeon S.M. de Mainetti, who also participated in the meeting.

### HPB MEETING BOLOGNA ITALY

The largest national chapter of the World Association is the Italian chapter. It has more than 200 members. To coincide with the 900 th anniversary of the University of Bologna, the Italian chapter of HPB surgery is arranging a large international meeting.

President of the congress is Professor G. Gozzetti and Dr. A. Principe is the general secretary. Among the guest speakers are F. Belzer, USA; S. Bengmark, Sweden, G. Benhamou, France; H. Bismuth, France; C. Broelsch, USA; J.L. Cameron, USA; C. Huguet, Monaco; B. Jeppsson, Sweden; P. Kestens, Belgium; J.M. Little, Australia; N.J. Lygidakis, Holland; E. Moreno Gonzales, Spain; G.B. Otte, Belgium; E. Passaro, USA; R.L. Rossi, USA; T.E. Starzl, USA; M.N. van der Heyde, Holland; A.L. Warsaw, USA; L. Wise, USA, in addition to numerous well-known Italian scientists. The meeting starts with an opening lecture with the title: HPB Surgery in the 90's, on November 13 in the afternoon. November 14 is mainly spent with discussions regarding focal lesions of the liver, progress in liver transplantation, cancer of the pancreas and endocrine tumors of the pancreas. On November 15 biliary surgery is extensively discussed: controversies and alternatives in managing of gallstones, intrahepatic stones, extracorporal stock-wave lithotripsy, gallstones in cirrhotics, surgical options in biliary stricture, and intraoperative ecography.
